# Transferrin-mediated iron sequestration suggests a novel therapeutic strategy for controlling *Nosema* disease in the honey bee, *Apis mellifera*

**DOI:** 10.1371/journal.ppat.1009270

**Published:** 2021-02-18

**Authors:** Cristina Rodríguez-García, Matthew C. Heerman, Steven C. Cook, Jay D. Evans, Gloria DeGrandi-Hoffman, Olubukola Banmeke, Yi Zhang, Shaokang Huang, Michele Hamilton, Yan Ping Chen

**Affiliations:** 1 USDA-ARS Bee Research Laboratory, Beltsville, Maryland, United States of America; 2 USDA-ARS Carl Hayden Bee Research Center, Tucson, Arizona, United States of America; 3 Guangdong Institute of Applied Biological Resources, Guangzhou, Guangdong Province, China; 4 College of Animal Sciences (Bee Science), Fujian Agriculture and Forestry University, Fuzhou, Fujian Province, China; ICIPE: International Centre for Insect Physiology and Ecology, KENYA

## Abstract

Nosemosis C, a *Nosema* disease caused by microsporidia parasite *Nosema ceranae*, is a significant disease burden of the European honey bee *Apis mellifera* which is one of the most economically important insect pollinators. Nevertheless, there is no effective treatment currently available for *Nosema* disease and the disease mechanisms underlying the pathological effects of *N*. *ceranae* infection in honey bees are poorly understood. Iron is an essential nutrient for growth and survival of hosts and pathogens alike. The iron tug-of-war between host and pathogen is a central battlefield at the host-pathogen interface which determines the outcome of an infection, however, has not been explored in honey bees. To fill the gap, we conducted a study to investigate the impact of *N*. *ceranae* infection on iron homeostasis in honey bees. The expression of transferrin, an iron binding and transporting protein that is one of the key players of iron homeostasis, in response to *N*. *ceranae* infection was analysed. Furthermore, the functional roles of transferrin in iron homeostasis and honey bee host immunity were characterized using an RNA interference (RNAi)-based method. The results showed that *N*. *ceranae* infection causes iron deficiency and upregulation of the *A*. *mellifera* transferrin (AmTsf) mRNA in honey bees, implying that higher expression of AmTsf allows *N*. *ceranae* to scavenge more iron from the host for its proliferation and survival. The suppressed expression levels of AmTsf via RNAi could lead to reduced *N*. *ceranae* transcription activity, alleviated iron loss, enhanced immunity, and improved survival of the infected bees. The intriguing multifunctionality of transferrin illustrated in this study is a significant contribution to the existing body of literature concerning iron homeostasis in insects. The uncovered functional role of transferrin on iron homeostasis, pathogen growth and honey bee’s ability to mount immune responses may hold the key for the development of novel strategies to treat or prevent diseases in honey bees.

## Author summary

*Nosema* disease is an adult honey bee disease caused by an intracellular parasite named *Nosema ceranae* that lives in the digestive tract of honeybees and is linked to colony losses worldwide. Iron is an essential nutrient for both pathogenic microbes and their hosts and relies largely on an iron transporter protein, transferrin. In this study, we found that *N*. *ceranae* infection could cause iron deficiency in infected bees. We also found that *N*. *ceranae* infection led to elevated levels of transferrin, allowing *N*. *ceranae* to scavenge more iron from the host for its survival. As a result, we employed a genetic method to interfere with the production of transferrin in *N*. *ceranae*-infected honeybees. The reduction of transferrin production and activity was found to accompany reduced iron loss and to improve the survival of *N*. *ceranae*-infected bees. This study suggests a novel strategy for bee disease treatment and should be of interest to researchers, graduate students, beekeepers and policymakers worldwide.

## Introduction

Pollinators are responsible for assisting the reproductive success of approximately 35 percent of global food crops and over three-fourths of the world's flowering plants [1]. The European honey bee *Apis mellifera* (Hymenoptera: Apidae) is the most widely managed pollinator in the world’s agricultural systems. In the United States alone, honey bees pollinate more than 80 percent of food crops and add more than $17 billion in economic value to agricultural production each year [2]. However, the health of honey bees have been declining in many parts of the world, threatening food security [3–6]. Among a wide range of possible factors that negatively impact the health of bees, infectious diseases caused by parasites and pathogens are significant threats to honey bees and other pollinators [7–10]. Nosemosis, or *Nosema* disease is caused by two species of obligate intracellular microsporidian parasites, *Nosema apis* and *N*. *ceranae*, and is one of the most common and severe adult honey bee diseases [11, 12]. For years, nosemosis in *A*. *mellifera* was thought to be caused by a single species of *Nosema*, *N*. *apis* which was first detected in *A*. *mellifera* in 1909 [13]. In 2005, natural infection of *N*. *ceranae*, a species of *Nosema* which was first described in the Asian honey bee *A*. *cerana* [14], was identified as a disease agent of *A*. *mellifera* [15]. Since its emergence, *N*. *ceranae* has been detected in *A*. *mellifera* colonies worldwide and is the more prevalent and virulent of two *Nosema* species [15–22]. Nosemosis type C [23], a *Nosema* disease caused by *N*. *ceranae*, has been associated with honey bee Colony Collapse Disorder (CCD) [4] and frequently implicated in heavy colony losses worldwide, particularly in the context of overwintering mortality [24–28].

Like any other microsporidia parasites, *Nosema* species produce environmentally resistant spores that are capable of surviving outside of their host for up to several years [25, 29, 30]. Honey bees become infected with *Nosema* when they ingest spore-contaminated food or water via trophallaxis or clean spore-contaminated combs via the fecal-oral route. The ingested spores germinate in the midgut lumen with a polar tubule extruding from a spore. The polar tubule pierces the membrane of the host’s midgut epithelial cell and serves as a conduit for delivering the infectious content, sporoplasm, directly into the host cell's cytoplasm. The vegetative stage of *Nosema* grows and multiplies inside epithelial cells and undergoes mergonic cycles and a sporogonic phase to produce mature spores that either infect adjacent cells or that are released into the midgut lumen via cell lysis and excreted in feces into the hive environment, thus providing new sources of infection. Through repeated multiplication, roughly 30 to 50 million spores accumulate inside a bee’s midgut within two weeks post-infection [29].

*Nosema* infection affects bee health and colony performance in multiple ways [11, 25]. The pathological effects of *Nosema* infection on honey bees include the impairment of various physiological functions; these include metabolic disorders, energetic stress, cognitive deficits, bodyweight reduction, and a decrease in life expectancy [30–35]. *Nosema* consumes the host’s metabolic resources for its own growth and proliferation, thereby imposing nutritional and energetic stress on honey bees and, in turn, hindering bees’ immunity. The weakened or compromised immune system of *Nosema* infected bees is evidenced by significant down-regulation of several immune molecules and the inhibition of apoptosis [36–40]. As a destructive intracellular parasite, *N*. *ceranae* not only causes serious disease in the form of nosemosis type C but also increases honey bees’ susceptibility to other pathogenic infections due to immunosuppression, leading to more complex and severe diseases [32, 41, 42]. At the colony level, infection with *N*. *ceranae* was reported as being associated with a decline in adult bee population size and brood rearing, a reduction in honey production, and an increase in queen supersedure and bee colony losses [17, 28, 29, 43, 44].

Despite more than a decade of research, the mechanisms underlying the high prevalence and pathological effects of *N*. *ceranae* infection in *A*. *mellifera* populations are still relatively poorly understood. Re-queening [45, 46], food supplements, and careful colony management [24, 47–52] are control measures that are currently available for managing *Nosema* disease. The only registered chemotherapy for *Nosema* disease is Fumagillin-B [53–57], a naturally secreted antibiotic of the fungus *Aspergillus fumigatus*. However, the use of Fumagillin in the treatment of bee diseases is banned in Europe and other countries as it has no established maximum residue level (MRL; European Commission Regulation). Fumagillin has also been linked to resistance development problems and off-target effects [58]. The problems associated with using Fumagillin has made the global crisis of honey bee population decline even direr. It is, therefore, imperative to improve our understanding of the molecular mechanisms of *Nosema* disease pathogenesis and host-pathogen interactions so that target- and mechanism-based therapeutic strategies that are less likely to develop resistance can be explored for effective disease treatment in honey bees.

Iron is an essential nutrient for the growth of both pathogens and their hosts [59, 60]. Therefore, limiting the iron availability of invading microbes during infection is an important component of the host’s innate immune response. Iron exists in two oxidation states, ferrous cation (Fe2+) and ferric cation (Fe3+), and therefore serves as a redox catalyst that either donates or accepts electrons, respectively. The redox potential of iron renders it readily available for use in diverse cellular processes; however, it can also lead to toxicity and cell death at excess levels via catalysis of the Fenton reaction generating free hydroxyl radicals that damage lipids, DNA, and protein. Therefore, the acquisition, transport, use, and storage of iron must be strictly regulated through a finely tuned mechanism to ensure iron’s essential nutritional benefits and to prevent its toxic effects [61, 62].

Transferrins are a group of iron-binding glycoproteins that have a molecular weight of roughly around 80 kDa and two homologous ferric-binding lobes, one each in the N- and C- termini (Liang et al., 1997). In mammals, transferrins are produced in the liver and are most abundant in blood and play a central role in iron homeostasis and transport for cellular uptake. Iron absorption occurs predominantly in the intestinal epithelial cells of the duodenum and proximal jejunum. Once ferric iron (Fe3+) is reduced to soluble ferrous iron (Fe2+) by ferrireductase on the intestinal brush border situated at the luminal pole of the enterocyte, Fe2+ is absorbed across the apical membrane of the intestinal enterocyte into the bloodstream via a protein called divalent metal transporter (DMT1 or NRAMP2). Immediately after in the bloodstream, iron is mostly bound by transferrins and transported to various cells and tissue. Cells take up iron by internalizing the transferrin-iron complex through transferrin receptor 1 (TfR1)-mediated endocytosis for iron to be released. The transferrin and TfR are recirculated into blood and cell membrane after iron release, respectively. Meanwhile, unused iron can be stored in an iron storage protein, i.e., ferritin. Under normal physiological conditions, virtually all iron in the body is tied up with ubiquitous transferrins and related proteins [63–65].

Progress in genomic sequencing and functional analysis has greatly advanced our understanding of insect iron metabolism and regulation. Previous studies have revealed mammalian homologous genes to be involved in iron metabolism in insect genomes and indicated that copies of transferrin genes can be found in the genomes of multiple insect species including *Drosophila melanogaster* (Diptera; fruit fly), *Apis mellifera* (Hymenoptera; honey bee), *Aedes aegypti* (Diptera; mosquito), *Antheraea Mylitta* (Lepidoptera; Silkworm), *Bombus ignitus* (Hymenoptera; bumble bee), *Apriona germari* (Coleoptera; mulberry longhorn beetle), and *Choristoneura fumiferana* (Lepidoptera; spruce budworm) [60, 66–69]. Based on the knowledge gained from the study of *Drosophila* species, a genetically tractable model organism, the insect transferrins have the same size as those of mammals with one or two ferric-binding lobes which can bind iron in one or both [60, 66, 68]. Among transferrin genetic variants identified in *Drosophila*, transferrin-1 is the only transferrin found in hemolymph, has the most significant homology to mammalian blood transferrin [70, 71], and plays a role in iron trafficking between the gut and the fat body which serves as a functional equivalent of the mammalian liver [68].

Insect transferrins are considered to be multifunctional proteins [67]. In addition to their role in dietary iron delivery, transferrins have additional functions that include serving as an antibiotic agent, an antioxidant, a vitellogenin, and a juvenile hormone-regulated protein [60, 67, 70, 72]. Previous studies in different orders of insects showed that the expression of transferrin-1 was upregulated in response to challenges presented by various infectious agents including bacteria, fungi, and parasites and that a loss of transferrin-1 function could result in increased availability of free iron while promoting oxidative stress [67], indicating antimicrobial properties of transferrin-1. Additionally, transferrin was shown to inhibit apoptosis by diminishing the Fenton reaction via the binding of free iron, illustrating a functional role of transferrin in reducing oxidative stress in insects [73]. Furthermore, transferrin was shown to function as a vitellogenic protein that is down-regulated by juvenile hormones and to transport iron for developing embryos, adding a new dimension to the functional role of transferrin in insects [67, 74].

In honey bees *A*. *mellifera*, a full-length cDNA encoding a putative transferrin (AmTsf) and which displays a high level of sequence conservation to other insect-derived transferrin was described and differentially expressed under a range of conditions including developmental stages, septic injury, and juvenile hormone treatment [75, 76]. The results showed that an elevated level of AmTsf transcript was found in the brain and compound eye of mature adult workers, in the early pupal stages and virgin queens [76]. The results also revealed that the transcriptional response of transferrin to septic injury with *E*. *coli* was relatively moderate compared to the expression of a gene encoding an antibacterial peptide, hymenoptaecin [76]. These studies indicated the broader multifunctional roles of transferrin in honey bees and prompted us to conduct research exploring the roles of this important iron metabolism protein in honey bees’ innate immunity to *Nosema* infection.

## Material and methods

### Ethics statement

The experimental honey bee colonies originated from package bees that were purchased from a commercial beekeeper in Florida were kept in the U.S. Department of Agriculture Agricultural Research Service (USDA-ARS) Bee Research Laboratory apiaries, Beltsville, Maryland, U.S. The apiaries are the property of the USDA-ARS and are not privately-owned or protected in any way. No specific permits were required for the described studies. The European honey bee (*Apis mellifera ligustica*) was used in the study and is neither an endangered nor protected species.

### Honey bee colonies

Honey bees were collected from colonies of *Apis mellifera ligustica* maintained at the USDA-ARS Bee Research Laboratory apiaries, Beltsville, Maryland, U.S. A monthly survey was carried out in these colonies to monitor the status of *N*. *ceranae* and virus infections by light microscope and molecular assays, following our previously described methods [77, 78]. Briefly, 30 foraging workers were collected at the hive entrance for each colony. The abdomens were removed from the foraging workers and ground up thoroughly in 30 ml of deionized H_2_O. 10 μl of the homogenate was loaded onto a hemocytometer (Neubauer-ruled Bright Line counting chambers; Hausser Scientific, Horsham, PA, USA). The presence of *Nosema* spores and the spore numbers were counted under light microscopy [79].

### Analysis of N. ceranae infected bees by natural infection under field conditions

Bee colonies were divided into three groups based on *N*. *ceranae* infection. *N*. *ceranae* infected colonies with below 1.2X 10^6^ spores / per bee (1.2M, N = 4 colonies) were defined as low-level of infection and *N*. *ceranae* infected colonies with more than 26 X 10^6^ spores / per bee (26M, N = 4 colonies) were defined as high-level of infection. Colonies that were free of *N*. *ceranae* infection were used as a negative control (N = 4 colonies). The concentration of the spore was determined using a hemocytometer under light microscopy as described above.

For each of the colonies, about 50 house or nurse bees were removed from the brood frame and about 50 forager bees were collected at the front of the hive. Ten nurse bees and ten forager bees from each of high-level and low-level *N*. *ceranae* infected colonies were used for RNA extraction. One step qRT-PCR assay was performed for measuring the transcriptional activity of *N*. *ceranae* 16S ribosomal RNA (rRNA) in both nurse bees and forager bees [78].

**Fig 1:**
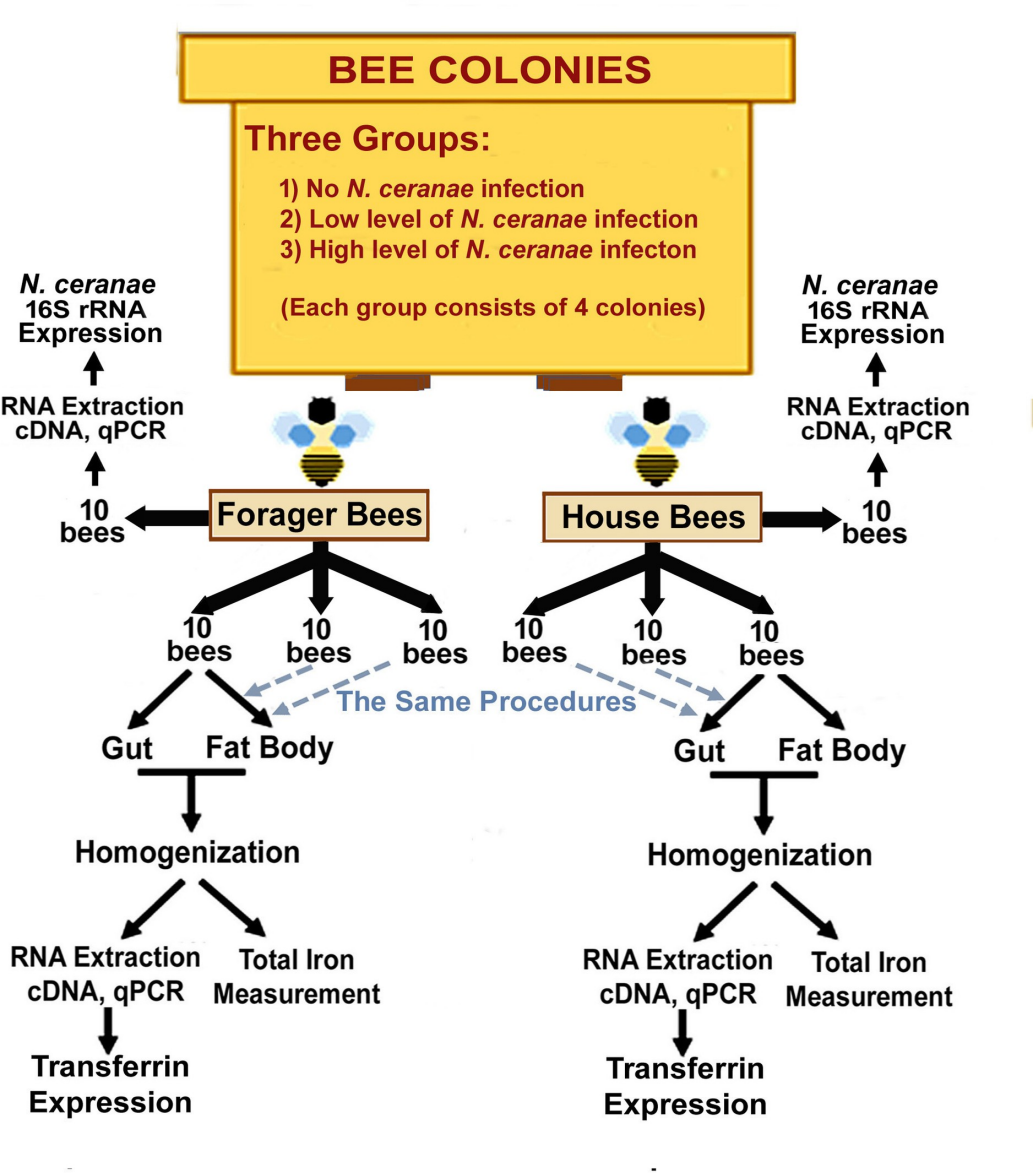
Graphic representation of the study design for field colonies.

Thirty nurse bees and thirty forager bees from each colony were used for subsequent transferrin expression analysis and total iron measurement. Both nurse bees and forager bees from the same colony were divided into three subgroups, each consisting of 10 bees. For each subgroup, tissues of the gut and fat body of individual bees were removed using a pair of fine forceps under a dissecting microscope, and placed separately in a 2 mL microcentrifuge tubes with a screw cap, containing sterile 1.4 mm zirconium silicate grinding beads (Quackenbush Co., Inc. IL, USA). Each type of tissue from the same subgroup was combined into one tube and disrupted in 1mL of homogenized buffer (1X tris buffered saline, 1ml/L protease inhibitor and 0.1ml/L non-denaturing detergent NP-40 (4-Nonylphenyl-polyethylene glycol at pH 7.24) using a FastPrep cell disrupter (Bio 101 Thermo Savant, Carlsbad, CA). After disruption, each tube was centrifuged at 10.000 g for ten minutes. Supernatant was collected for iron measurement and RNA extraction. RT-qPCR was performed to measure the gene expression level of transferrin (AmTsf). The experimental design is illustrated in **Fig 1.**

### Nosema inoculum preparation, laboratory cage assay setup, and data collection

To prepare *Nosema* inoculum, forager honey bees were collected with an insect vacuum outside the hive entrance from colonies that were identified as *N*. *ceranae*-positive. Individual bees were chilled on ice until immobile and then the midguts were removed from the live bees by using a pair of forceps and pulling on the last abdominal segment of the bee until the midgut was separated from the bee abdomen. Approximately 300 midguts were collected and homogenized in sterile, distilled water. The homogenate was filtered through a 0.65μM nylon mesh cloth (EMD Millipore) to remove tissue debris and then centrifuged at 3,000×g for five minutes. The supernatant was discarded and the spore pellet was washed in sterile water and centrifuged for 10 minutes at 5,000×g. The washing step was repeated two more times. The concentration of purified spore was determined using a hemocytometer and diluted to a concentration of 2.0×10^7^ spores/ml in 50% (w/v) sucrose solution.

**Fig 2:**
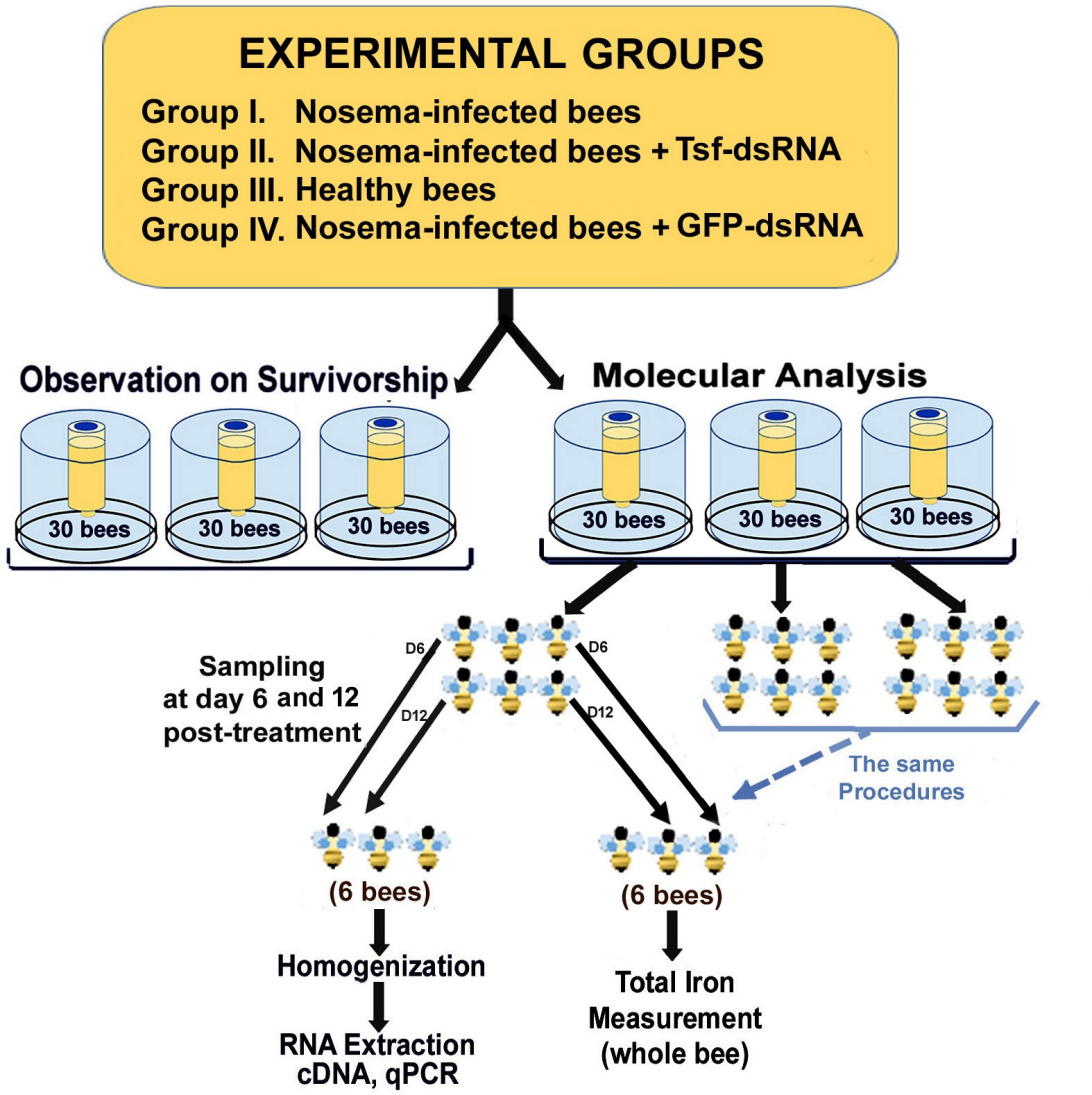
Graphic representation of the study design for laboratory cage assay.

The experimental design for the laboratory cage study is illustrated in **Fig 2**. To set up laboratory cages, frames with sealed brood from healthy colonies identified as *Nosema*-negative by our monthly disease survey were removed from bee colonies and placed individually in a mesh-walled cage and incubated in an insect growth chamber at 34±1°C, 55±5% RH overnight. After 24 hours to allow newly emerged bees to roam on the whole frame consisting of brood, pollen, and honey to acquire the necessary gut microbiota, the newly emerged worker bees were collected and mixed for subsequent *N*. *ceranae* inoculation. The bees were starved for at least 2 hours before the subsequent *Nosema* inoculation.

Individual starved bees were fed with 2μL of inoculum solution (5 X 10^4^ spores/bee) via P10 pipette and then transferred into a rearing cage [80, 81]. Uninfected bees (negative control) were fed with 2μL of 50% sucrose syrup. The inoculated and control bees were transferred separately to the rearing cages. The caged bees under controlled laboratory conditions were divided into three groups based on the treatment they received: Group I. *Nosema*-infected bees (infected/untreated), Group II. *Nosema*-infected bees + AmTsf-dsRNA treatment, Group III. Healthy bees (uninfected/untreated) and Group IV Nosem-infected bees + Green Fluorescent Protein (GFP)-dsRNA treatment. The bees in Group II were fed with 20 ng/ml AmTsf-dsRNA diluted in 50% sugar solution ad libitum from a 3mL syringe. The bees in Group IV were fed with 20 ng/ml GFP-dsRNA diluted in 50% sugar solution ad libitum from a 3mL syringe. The untreated bees and healthy bees were fed with only a 50% sugar solution. The solution for each group was changed every three days. A piece of pollen patty was also supplied at the bottom of each cage to provide proteins, lipids, and sterols. The sucrose solution was renewed every two days and the pollen patty was changed every three days. All cup cages were maintained in an insect incubator (32°C, 75% RH).

Each treatment group consisted of six cages ((30 bees/cage), three cages for molecular analysis and total iron quantification, and the other three for observation on bee survivorship. The number of dead bees was recorded and removed daily. Bees were sampled at days 6, and 12 post-treatment. At each time point post-treatment, six bees were sampled from each cage (6 X 3 cages = 18 bees). Nine bees were used for total iron measurement and nine bees were used to obtain tissues of gut and fat body following the methods described above. Dissected tissues were subjected to RNA extraction. RT-qPCR was performed for the measurement of gene expression of *N*. *ceranae* 16S rRNA, transferrin (AmTsf), immune transcripts (*Abaecin*, and *Apidaecin*), BIRC5, a negative regulator of cell apoptosis, and two core components of RNAi pathway, *Dicer* and *Argonaute*. The qRT-PCR primers used for detecting AmTsf transcript were designed to ensure they were located outside of the dsRNA fragment.

### Molecular and biochemical methods

#### Primers

Table 1 shows the list of all primers used in this study. For transferrin gene (*AmTsf*), primers for RT-qPCR detection and dsRNA synthesis were designed from the conserved regions of *A*. *mellifera* transferrin (*AmTfr*) published by do Nascimiento et al. [75] (Genebank accession # AY336529) based on the alignment of homologous sequences. The sequence of green fluorescent protein vector (GFP, Clontech) was used as a non-target control. E-RNAi web service was used to design and evaluate long dsRNAs [82]. Primers were designed using the Primer3 web tool (http://bioinfo.ut.ee/primer3-0.4.0/). Primers for the production of dsRNA were fused with a T7 promoter sequence (5’-taa tac gac tca cta tag ggc ga-3’).

**Table 1:** Primers used in the present study.

Purpose	Gene Name	Primer	Sequence (5´—3´)	Reference	Length (bp)	qPCR primers data
dsRNA	***Tsf***	Tsf-Fw	taatacgactcactatagggagaGGAAAATAACGCGGATTTGA	This study–[75] Genebank accession # AY336529	482	

	*(Apis mellifera)*	Tsf-Rv	taatacgactcactatagggagaGAACGCTACGTCTCCTTTGC			
	***GFP***	GFP-Fw	TAATACGACTCACTATAGGGCGATTCCATGGCCAACACTTGTCA	(Li et al., 2016)		
			
		GFP-Rv	TAATACGACTCACTATAGGGCGATCAAGAAGGACCATGTGGT			
qPCR	***Tsf***	Tsf-Fw-qPCR	TCCGACATCGGAAAACCCAG	This study	100	E = 109.1% R^2 = 0.995 Slope = -3.122 y-int = 27.518
	*(Apis mellifera)*	Tsf-Rv-qPCR	CAGCCCAAGTGCATGGTTTC			
	***16S rRNA***	rRNA-F	CGGATAAAAGAGTCCGTTACC	(Chen *et al*., 2008)		
	*(N*.*ceranae)*	rRNA-R	TGAGCAGGGTTCTAGGGAT			
	***ß-actin***	ß-actin-F	ATGCCAACACTGTCCTTTCTGG	(Yang and Cox-Foster, 2005)		
	*(A*.*mellifera)*	ß-actin-R	GACCCACCAATCCATACGGA			
	***Abaecin***	Abaecin-F	CAGCATTCGCATACGTACCA	(Evans *et al*., 2006)		
	*(A*.*mellifera)*	Abaecin-R	GACCAGGAAACGTTGGAAAC			
	***Argonaute***	AGO-F	AGCCTTTAGAACTCTTGCTGGT	This study	96	E = 105.1% R^2 = 0.994 Slope = -3.205 y-int = 33.145
	*(A*.*mellifera)*	AGO-R	ACCTGCTGAGTTATGCACAGT	Genebank accession XM_395048.6		
	***Dicer***	DIC-F	AATGTTGGGTGAGAGCATGA	This study	94	E = 104.9% R^2 = 0.992 Slope = -3.209 y-int = 24.805
	*(A*.*mellifera)*	DIC-R	TGCTTGTTCAAGTTCCTTTGG	Genebank accession NM_001123013.2		
	***Apidaecin***	Apidaecin-F	TTTTGCCTTAGCAATTCTTGTTG	(Li *et al*., 2016)		
	*(A*.*mellifera)*	Apidaecin-R	GCAGGTCGAGTAGGCGGATCT			
	***birc5***	birc5-F	CTTCTGACAATTCGTGCAATCC	(Martín-Hernández *et al*., 2017)		
	*(A*.*mellifera)*	birc5-R	GGGTTCTTTCTTACCACCCACTAC			

#### RNA extraction and cDNA synthesis

TRIzol (Thermo Fisher Scientific, Waltham, MA, USA), a ready-to-use reagent for the isolation of high-quality total RNA, was added to each tube to extract total RNA from tissues of gut and fat body individually, following the manufacturer's instructions. DNase I (Thermo Fisher Scientific) was applied to remove any genomic DNA contamination. The resultant RNA pellets were resuspended in DNase- and RNase-free water (Thermo Fisher Scientific) in the presence of ribonuclease inhibitor (Thermo Fisher Scientific). The concentration and purity of RNA were measured using a NanoDrop 8000 spectrophotometer (Thermo Fisher Scientific). All RNAs were stored at -80°C until used. First-strand cDNA was synthesized from extracted RNA using the Retrotranscriptase kit (Thermo Fisher Scientific) according to the manufacturer’s protocol and used for the subsequent qPCR assays.

#### qPCR

Quantitative real-time PCR (qPCR) were run on a CFX384 Touch Real-Time PCR System (Bio-Rad, Hercules, CA). SYBR Green was used as a dye for quantitative PCR and product melting analysis. *Apis mellifera* ß-actin was used as a reference gene to normalize the raw gene expression data. Each 10μL PCR mixture was assembled by mixing 5μL 2× Brilliant II Ultra-Fast SYBR Green qPCR mix (Agilent Technologies, Santa Clara, CA, USA), 0.25μL forward primer (20mM) reverse primer, 0.5μL cDNA, and 4μL nuclease-free water. Each reaction was run in triplicate. Positive and negative controls were run in parallel. The qPCRs were performed at 95°C for 3min, followed by 40 cycles of 95°C for 10s and 60°C for 45s. Amplification efficiency and melting curves were monitored to evaluate the quality and specificity of amplification. The comparative Ct method (2-^ΔΔCt^) method [82] was used for calculating the relative expression of various transcripts mentioned above among different treatment groups.

#### Iron quantification

Total iron (Fe2+ and Fe3+) was measured using a commercial colorimetric kit following the manufacturer’s instructions (Iron Colorimetric Assay kit, BioVision Inc. Milpitas, CA). The absorbance was measured at 593 nM (BioTek Synergy H1).

#### dsRNA synthesis

An aliquot of the cDNA of an adult bee was used for the amplification of AmTsf. Conventional PCR reactions were performed to obtain enough DNA template. PCR reaction mixtures of 100μL contained the following components: 78μL H_2_O, 10μL 10× reaction buffer (Invitrogen), 3μL MgCl_2_, 2μL dNTP mix (10mM, Invitrogen), 2μL forward primer (20μM), 2μL reverse primer (20 μM), 1μL Taq polymerase (Invitrogen), and 2μL DNA template. The thermal profile of the PCR amplification was as follows: one cycle of 94°C for 3 min followed by 35 cycles of 94°C for 30 sec, 56°C for 30 sec, 72°C for 90 sec with a final extension of 72°C for 10 min. After PCR amplification, gel electrophoresis via 1.0% agarose gels was performed to verify the expected target. The purified PCR product was used as a template for the *in vitro* transcription reaction. The dsRNA was synthesized using the MEGAscript RNAi Kit (Ambion/ Thermo Fisher Scientific, Waltham, MA, USA) following the manufacturer’s instructions. The products of AmTsf -dsRNA and GFP-dsRNA were verified in 1.0% agarose gels and the concentration of dsRNA was determined with NanoDrop 8000 spectrophotometer (Thermo Fisher Scientific, Waltham, MA, USA). The *A*mTsf-dsRNA and GFP-dsRNA were diluted to the concentration of 20ng /μL in 50% sucrose solution based on a pilot study to define the effective concentration of dsRNA.

### Data analyses

After confirmation of a normal distribution and an equal variance of data, comparisons in transcriptional activity of *N*. *ceranae* 16S ribosomal RNA (rRNA) during the infection were made between the nurse bees and forager bees using a student’s t-test. A one-way analysis of variance (ANOVA) was used to determine differences in the amount of total iron and the expression level of AmTsf among different colony groups including high-level *N*. *ceranae* infection, low-level *N*. *ceranae* infection, and free of infection for guts and fat bodies of the nurse bees, and guts and fat bodies of the forager bees. The amount of total iron and the expression level of AmTsf between guts and fat bodies and between nurse bees and forager bees were compared using a Student’s t-test.

For analysis of *N*. *ceranae* infected bees under controlled laboratory conditions, the one-way ANOVA was also used to determine whether there were any statistically significant differences between different experimental groups. The experimental groups included I) *Nosema*-infected bees, II) *Nosema*-infected bees + AmTsf-dsRNA treatment, III) healthy bees (uninfected bees), and IV) *Nosema*-infected bees + GFP-dsRNA control regarding i) amount of total iron, ii) expression of AmTsf, iii) expression of immune transcripts *Abaecin*, and *Apidaecin*, iv) expression of cell apoptosis inhibitor, BIRC5, and v) expression of core components of RNAi pathway, *Dicer* and *Argonaute*. The Tukey's honestly significant difference test (Tukey's HSD) was used to test all pairwise differences among sample means for significance. Survival analysis was performed using the Kaplan-Meier method, and log-rank and Wilcoxon tests were used to assess the impact of AmTsf-dsRNA treatment on the improvement on the survivorship of Nosema-infected bees in comparison to Nosema-infected bees without treatment and healthy bees.

In all cases, a *p*-value of ≤ 0.05 was taken to be significant. All analyses were carried out using SPSS software 18.0 (Cary, NC, USA) and all the figures were generated using GraphPad Prism 7 (GraphPad Software, Inc., San Diego, CA, USA).

## Results

### Forager bees displayed a significantly higher level of N. ceranae 16S rRNA expression than nurse bees from the same colonies

**Fig 3:**
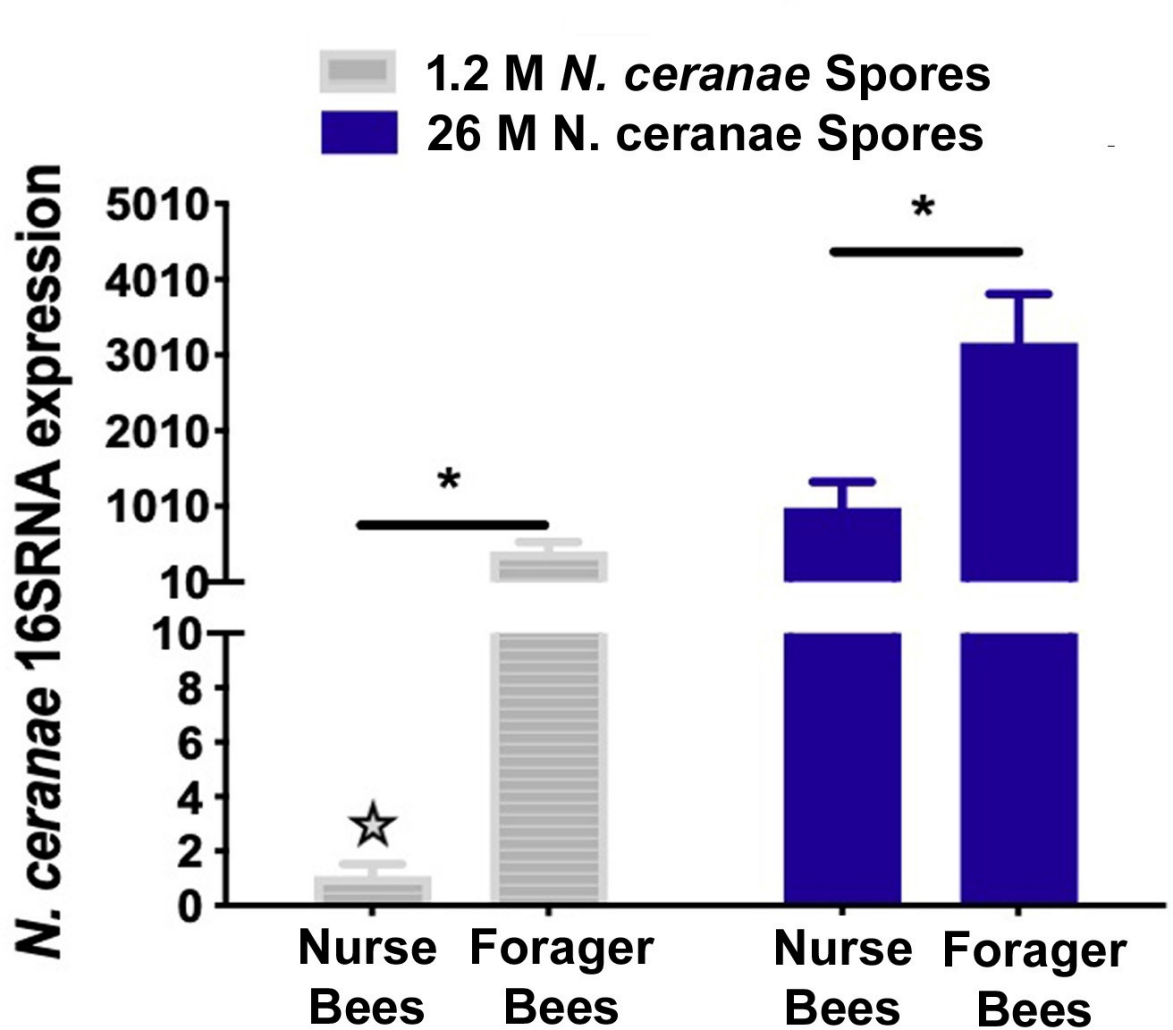
Expression levels of *N*. *ceranae* 16S rRNA. The transcriptional activity of *N*. *ceranae* 16S rRNA was corresponding with the level of spore loads in infected bees. The relative expression of *N*. *ceranae* 16S rRNA in forager bees was significantly higher than in nurse bees under both high- and low- levels of infection (1.2 M vs 26 M *N*. *ceranae* spores per bee). The relative expression was expressed as an n-fold difference relative to the calibrator (marked by a star) by 2^–∆∆Ct^ method. A star denotes a calibrator and an asterisk (*) denotes a statistically significant difference between the two groups (*P* ≤ 0.05, Student’s t-test).

Ribosome biogenesis is a central process in microbial growth. The activity of *N*. *cerana*e during the infection was demonstrated by the transcriptional activity of 16S ribosomal RNA (rRNA) in honey bees. The relative abundance of 16S rRNA gene expression by RT-qPCR analysis showed that there was good agreement between the *N*. *ceranae* spore loads (1.2 M *N*. *cerana*e spores vs 26 M *N*. *cerana*e spores) and the 16S rRNA expression level in infected bees. The higher the *N*. *ceranae* spore load, the higher the level of 16S rRNA expression detected in infected bees. The result also showed that forager bees had a significantly higher level of *N*. *ceranae* transcriptional activity than nurse bees from the same colonies under both high- and low- levels of the infection [26M:, df = 2, 1.2M:, df = 2;, P = 0.0284, P = 0.0483, Student’s, t = 3957, t = 5.806, t-test.] (**Fig 3**).

### N. ceranae infection resulted in a decrease in the amount of total iron and an increase in the level of transferrin expression in honey bees

**Fig 4:**
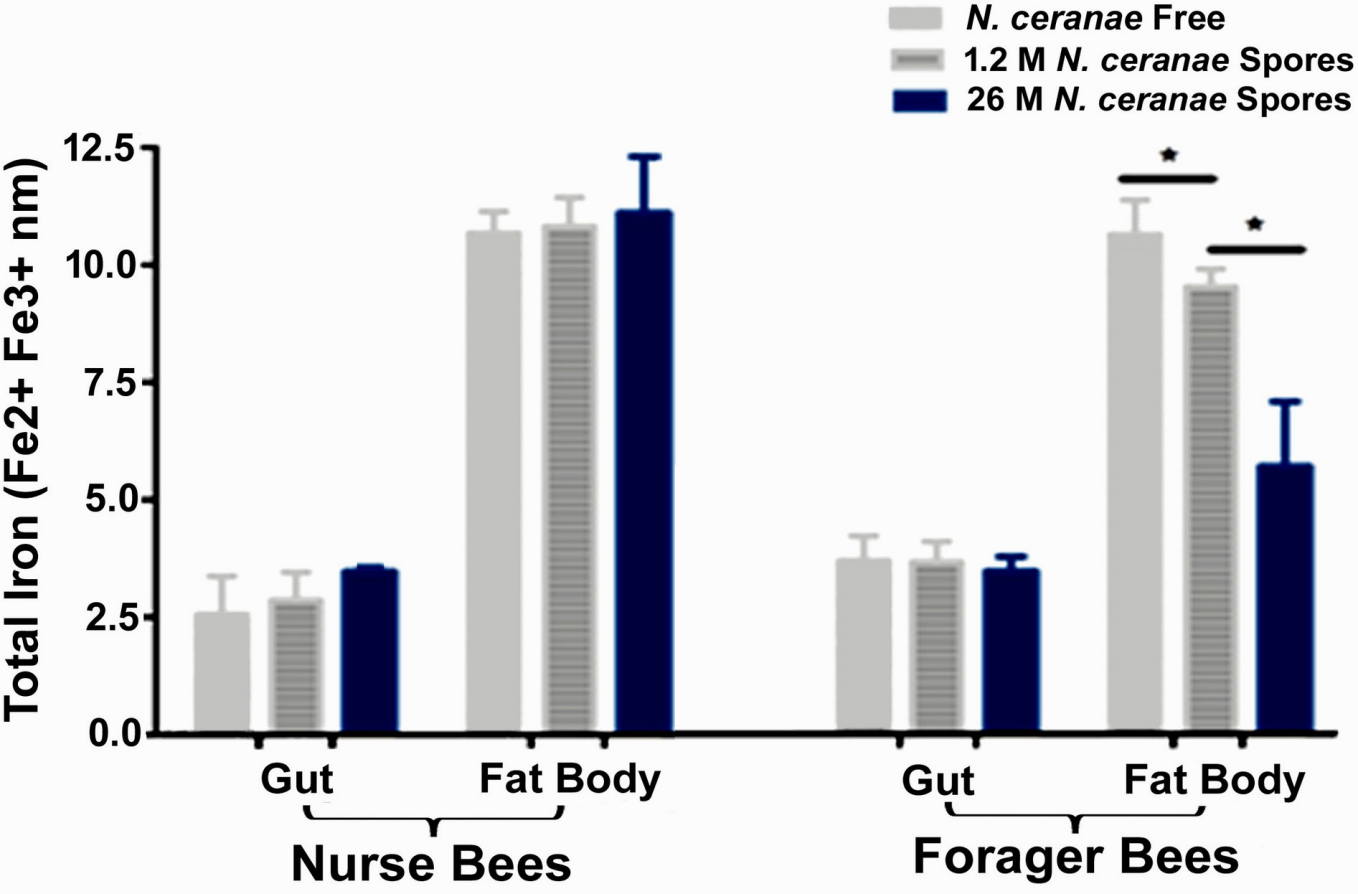
Tissue specific total amount of iron in gut and fat body tissues of nurse and forager bees. An asterisk (*) denotes a statistically significant difference between the two groups (p ≤ 0.05).

The measurement of ferrous and/or ferric ions showed that the total amount of iron was significantly higher in fat bodies than in guts for both nurse bees and forager bees, suggesting that iron was deposited predominantly in the fat bodies after absorption from foods by the intestinal epithelia. While there is no significant difference in total iron in the gut and fat body of nurse bees and in the gut of foraging bees, there was a significant difference in the total amount of iron in the fat body of foraging bees among different groups (*P* ≤ 0.0016, F = 22,48, one-way ANOVA). A significant *Nosema* dose-dependent reduction of the iron levels was found in the fat bodies of forager bees, with the lowest level of the total iron observed in the high-level *N*. *ceranae* infected colonies, followed by the low-level *N*. *ceranae* infected colonies and non-infected colonies [Tukey multiple comparisons. *N*. *ceranae* free vs. 1.2 M *N*. *ceranae* spores: *P* = 0.048357; *N*. *ceranae* free vs. 2.6 M *N*.*ceranae* spores: *P* = 0.0022; 1.2 M *N*. *ceranae* spores vs. 2.6 M *N*. *ceranae* spores: *P* = 0.0037] (**Fig 4**).

**Fig 5:**
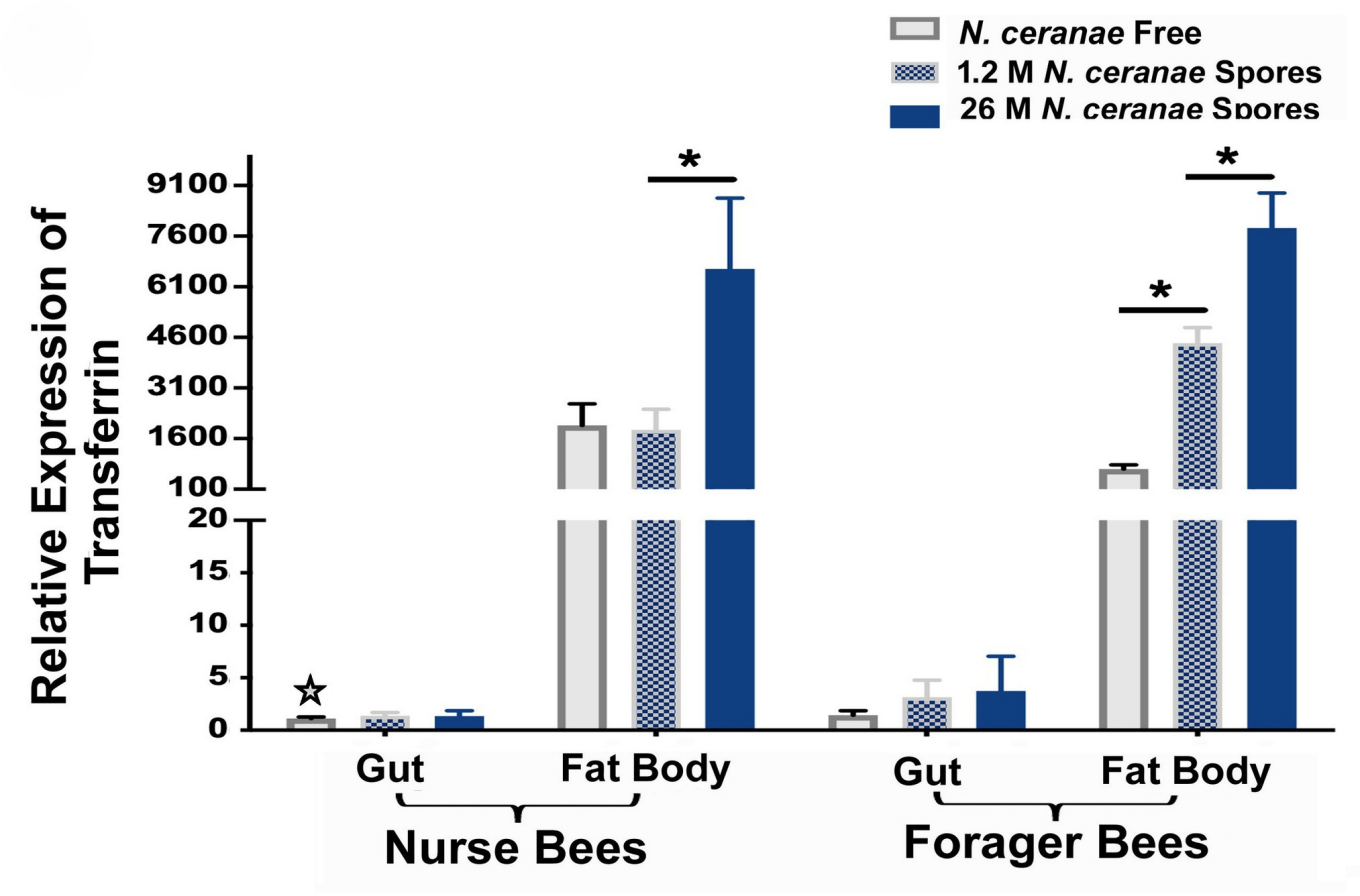
Tissue-specific relative transferrin mRNA transcript level in gut and fat body tissues of nurse forager bees. The relative expression was expressed as an n-fold difference relative to the calibrator (marked by a star) by 2^–∆∆Ct^ method. A star denotes a calibrator and an asterisk (*) denotes a statistically significant difference between the two groups (*P* ≤ 0.05).

Tissue specific AmTsf mRNA transcription profiles were generated for the guts and fat bodies of nurse bees and forager bees (**Fig 5**). While the difference in the expression level of AmTsf in the tissue of guts of both nurse bees and forager bees was insignificant, the transcription levels of AmTsf were significantly upregulated in the fat bodies of infected bees [fat bodies nurse bees: *P* = 0.006, F = 12,9; Fat bodies forager bees: *P* ≤ 0.0001, F = 88.9, one-way ANOVA]. The expression level of AmTsf in the fat bodies of forager bees was positively correlated with the *N*. *ceranae* loads; high spore loads generated high transcript expression levels of AmTsf [Tukey multiple comparisons, *N*. *ceranae* free vs. 1.2 M *N*.*ceranae* spores: *P* = 0.0011; *N*. *ceranae* free vs. 2.6 M *N*.*ceranae* spores: *P* <0.0001; 1.2 M *N*.*ceranae* spores vs. 2.6 M *N*.*ceranae* spores: *P* = 0.0017]. There was an inverse relationship between the expression level of the AmTsf transcript and total iron in the fat bodies of forager bees (Fig 4 and Fig 5).

### Ingestion of AmTsf -dsRNA knocked down the transcript level of the transferrin gene, leading to a reduction of N. ceranae levels

**Fig 6:**
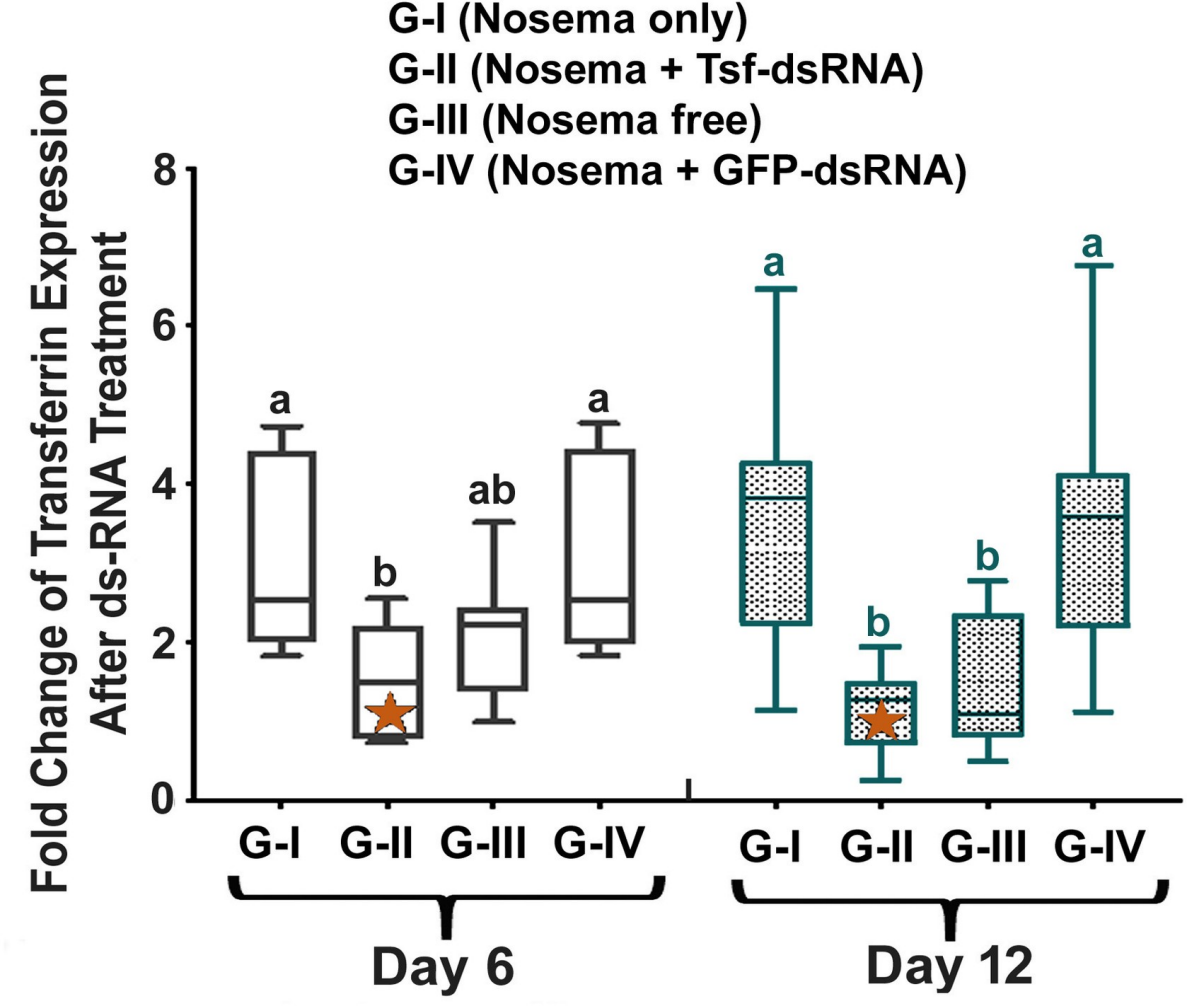
The relative expression of t*ransferrin* after treatment of *AmTsf* -dsRNA. The relative expression was expressed as an n-fold difference relative to the calibrator (marked by a star) by 2^–∆∆Ct^ method. A star denotes a calibrator and the different lower-case letters above bars indicate the statistically significant difference among different groups (*P* ≤ 0.05, ANOVA and Tukey´s tests).

Ingestion of AmTsf -dsRNA resulted in a significant reduction of AmTsf transcript levels 6 and 12 days post treatment (Group II) compared to Group I of *N*. *ceranae* infected bees without treatment and Group III of healthy bees, confirming the target specificity of the RNAi treatment [6 day: *P* = 0.0057, F(3,30) = 5.103, One-way ANOVA and Tukey's multiple comparisons test, G-I vs. G-II *P = 0*.*00255*, G-II vs. G-IV *P* = 0,7218; 12 day: *P* = 0.0002, F(3,30) = 9.294, One-way ANOVA and Tukey's multiple comparisons test G-I vs. G-II, *P* = 0.0017, G-II vs. G-IV *P =* 0,9023] (**Fig 6**).

Concomitantly, *N*. *ceranae* infection was significantly reduced after the ingestion of AmTsf-dsRNA from day 6 to day 12 in Group II, compared to bees in Group I (infected and untreated)

**Fig 7:**
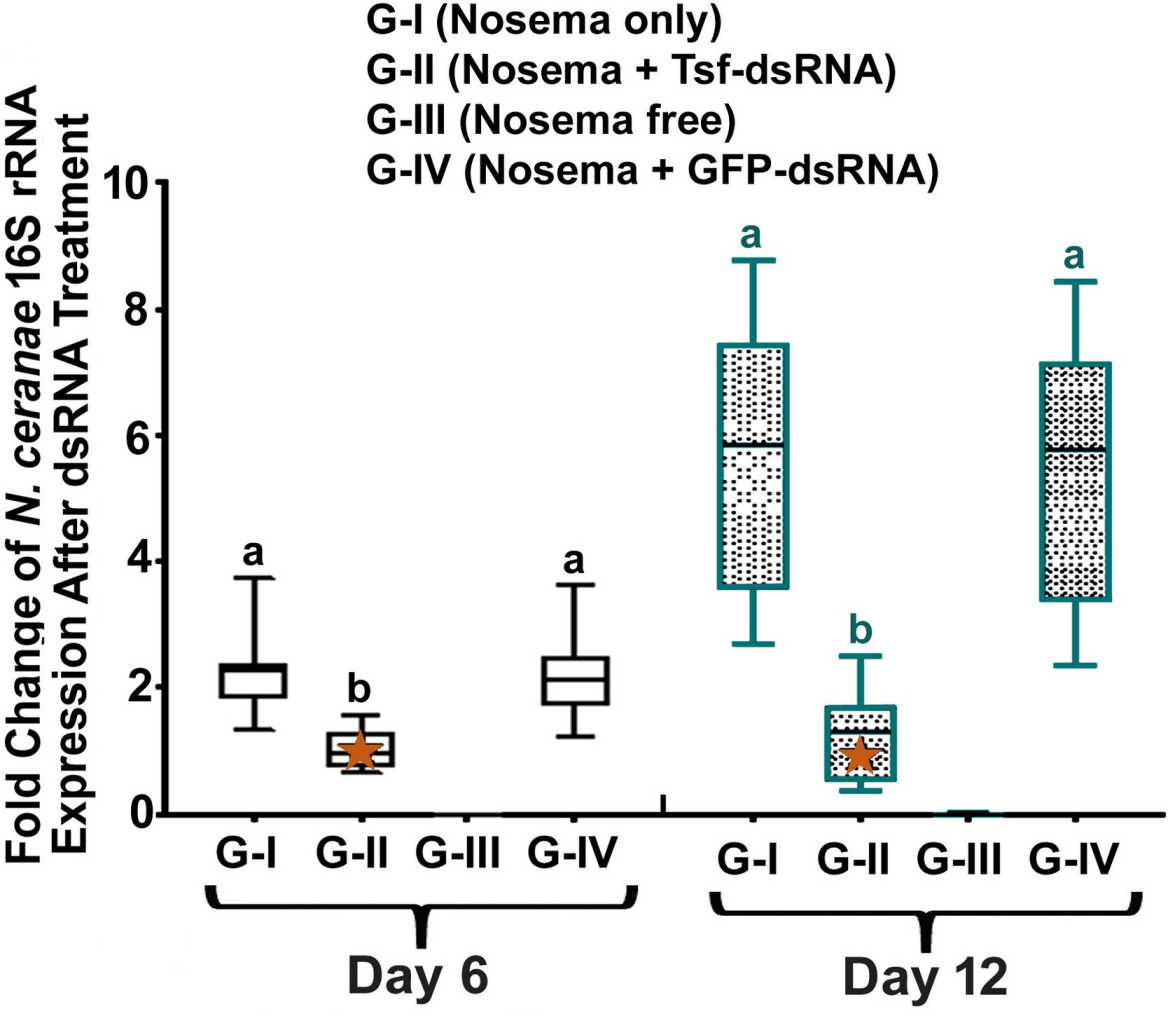
The relative expression of *N*. *ceranae* 16S rRNA after treatment of *AmTsf* -dsRNA. The relative expression was expressed as an n-fold difference relative to the calibrator (marked by a star) by 2^–∆∆Ct^ method. A star denotes a calibrator and the different lower-case letters above bars indicate the statistically significant difference among different groups (*P* ≤ 0.05, ANOVA and Tukey´s tests).

[6 day: *P*<0.0001, F(3,32) = 39.36, One-way ANOVA and Tukey's multiple comparisons to test different letters mean statistical significance, G-I vs. G-II *P* = 0,0001, G-II vs. G-IV *P* = 0,0002; 12 day: *P*<0.0001, F(3,32) = 29.87, One-way ANOVA and Tukey's multiple comparisons test G-I vs. G-II, *P*<0.0001, G-II vs. G-IV *P* <0,0001] (**Fig 7**).

### Ingestion of AmTsf-dsRNA alleviated the loss of total iron in N. ceranae infected bees in a time-dependent manner

**Fig 8:**
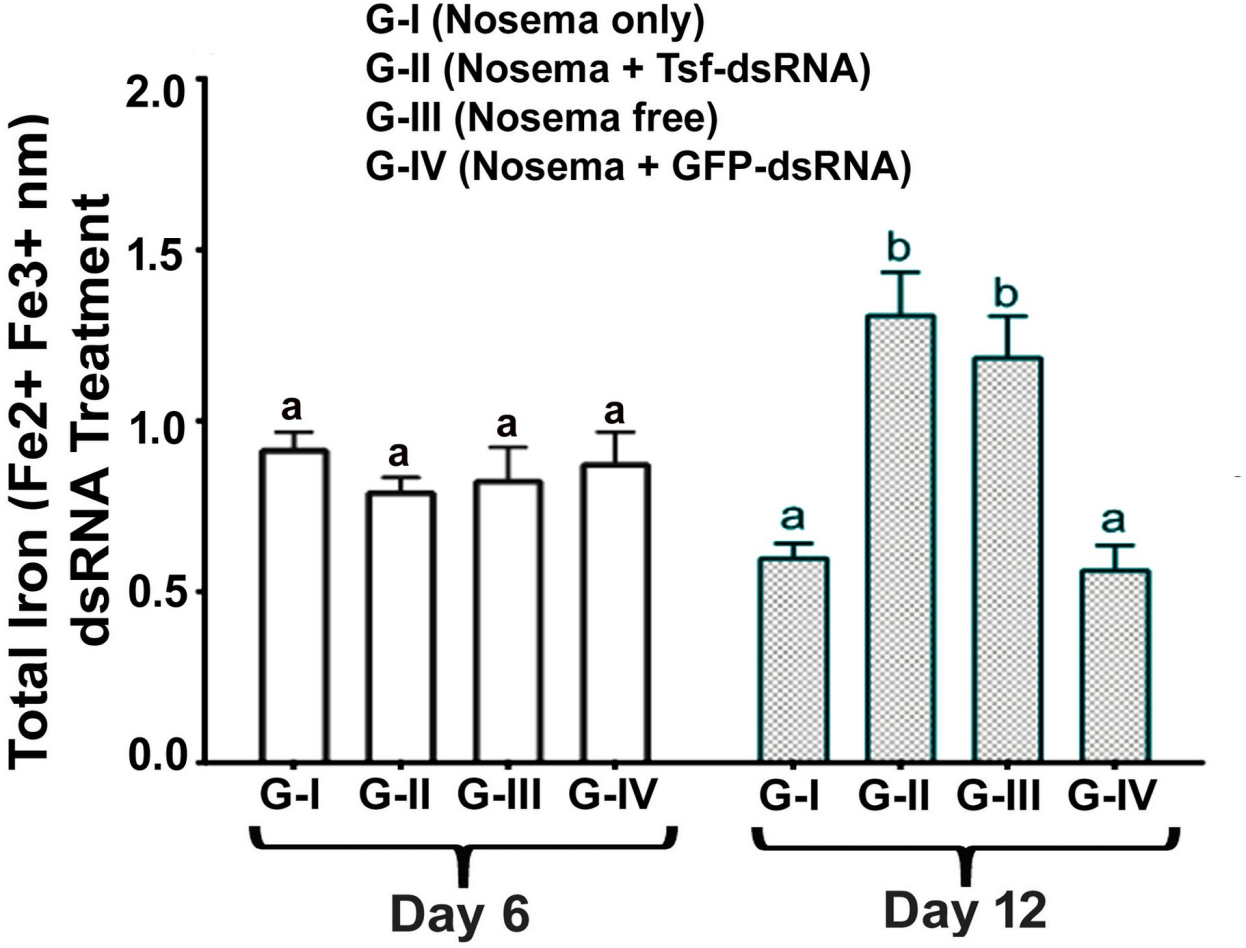
Total amount of iron in bees on day 6 and day 12 post AmTsf -dsRNA treatment. A star denotes a calibrator and the different lower-case letters above bars indicate the statistically significant difference among different groups (*P* ≤ 0.05, ANOVA and Tukey´s tests).

The temporal progression of *N*. *ceranae* infection caused significant loss of iron over time in honey bees is demonstrated by the result that the total amount of iron at day 12 was lower than that at day 6 post *N*. *ceranae* inoculation in Group I of the Nosema infected bees, illustrating progressive iron loss during the infection (**Fig 8**). Excitingly, the ingestion of AmTsf -dsRNA was able to ameliorate the loss of total iron caused by *N*. *ceranae* infection in a time-dependent manner. While there was no significant difference in the amount of total iron between Group I and Group II on day 6, the total amount of iron at day 12 in infected bees (Group I) was significantly lower than in infected bees from Group II that were treated with AmTsf-dsRNA and Group III of uninfected healthy bees as well [6 day: *P* = 0.218, F = 1,841, One-way ANOVA: 12 day: *P*<0,0001, F = 53,41, one-way ANOVA and Tukey's multiple comparisons test G-I vs. G-II *P*<0,0001, G-II vs. G-IV *P<0*,*0001*] (**Fig 8**).

### Silencing of the AmTsf transcript level led to increased immune transcription levels and decreased apoptotic inhibition

**Fig 9:**
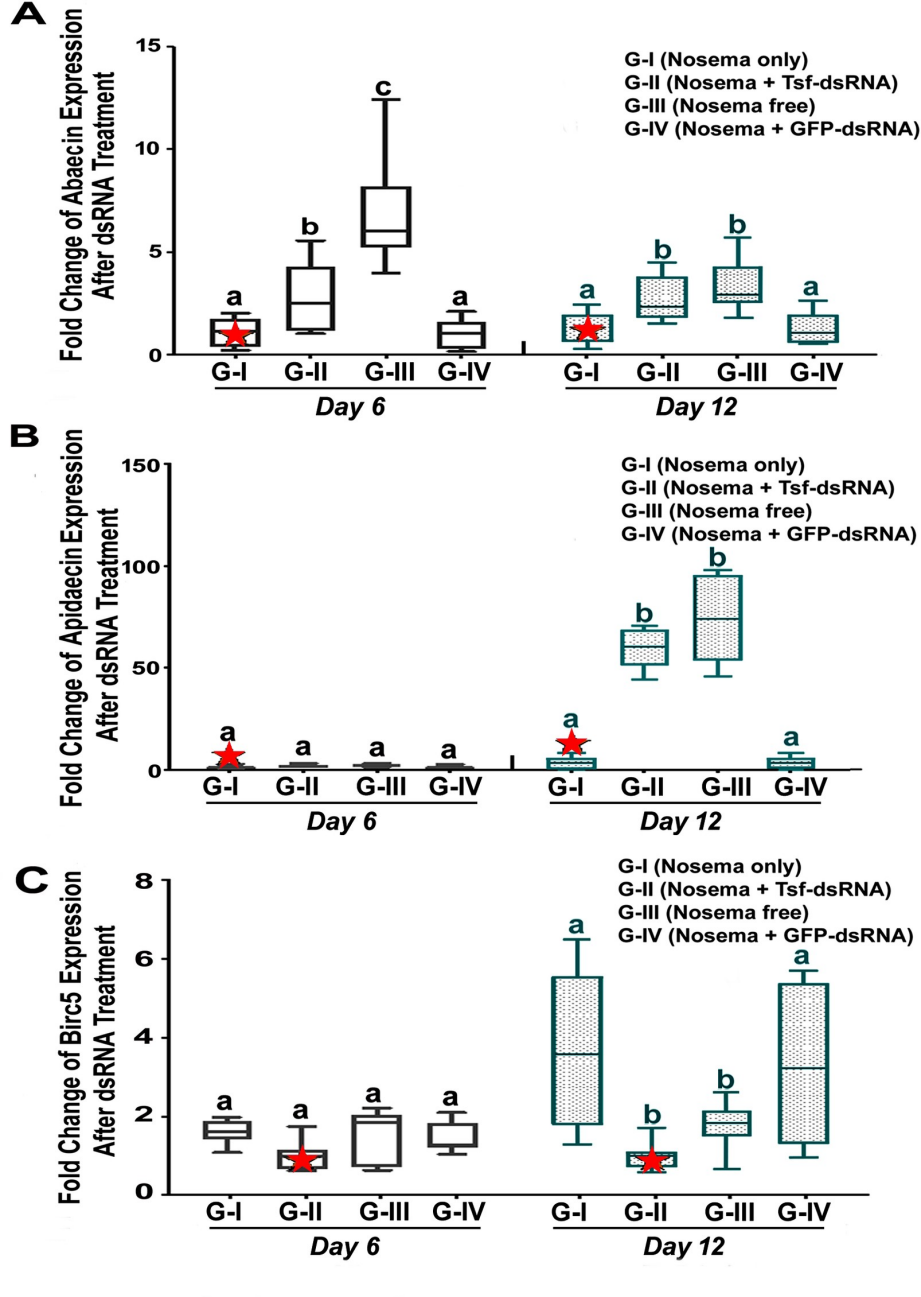
Time dependent transcription profiles for genes encoding antimicrobial immune peptides. (A) *Apidaecin*, (B) *Abaecin*, and (C) for gene encoding inhibitor of apoptosis *Birc5*. The relative expression was expressed as an n-fold difference relative to the calibrator (marked by a star) by 2^–∆∆Ct^ method. A star denotes a calibrator and the different lower-case letters above bars indicate the statistically significant difference among different groups (*P* ≤ 0.05, ANOVA and Tukey´s tests).

The expression of genes encoding antimicrobial peptides *apidaecin* and *abaecin* was used to measure the AmTsf-dsRNA induced immune responses in *N*. *ceranae* infected bees. In addition, the transcript level of a gene encoding a member of the inhibitor of apoptosis gene family, BIRC*5* (baculoviral IAP repeat-containing 5), was also used to measure immune response in *N*. *ceranae* infected bees post AmTsf-dsRNA treatment. As shown in **Fig 9**, while a*pidaecin* and *abaecin* transcript levels were downregulated in *N*. *ceranae* infected bees (Group I) compared to healthy bees (Group III), the AmTsf-dsRNA treatment could rescue and elevate the expression levels of genes encoding *apidaecin* and *abaecin* (Group II) [Abaecin, 6 day: *P*<0.0001, F(3,25) = 18.33 One-way ANOVA and Tukey´s multiple comparisons test G-I vs. G-II *P≤*0.00001, G-II vs. G-III *P* = 0.0009, G-II vs. G-IV P = 0,1666; G-III vs. G-IV *P*<0.0001; 12 day: *P* = 0.001, F(3,31) = 9.635, One-way ANOVA and Tukey's multiple comparisons test G-I vs. G-II P = 0.0235, G-II vs. G-IV P = 0,0383; Apidaecin, 12 day: *P* = 0.0001, F(3,28) = 88.04, One-way ANOVA and Tukey's multiple comparisons test G-I vs. G-II; P ≤ 0.0001, G-I vs G-III P<0,0001, G-II vs. G-III P = 0.4738, G-II vs G-IV P<0,0001, G-III vs. G-IV P<0,0001]. While the expression of genes encoding BIRC5 was upregulated in Group I of *N*. *ceranae* infected bees, the treatment with AmTsf-dsRNA helped alleviate the suppressive effect of BIRC5 gene, as shown by a significant reduction of BIRC5 expression level in Group II at day 12 post-treatment [12 day: *P* = 0.0014, F(3,28) = 6.828, One-way ANOVA and Tukey's multiple comparisons test G-I vs. G-II; P = 0.004, G-II vs. G-IV P = 0,0069] (**Fig 9**).

### RNAi pathway was activated post AmTsf -dsRNA treatment

**Fig 10:**
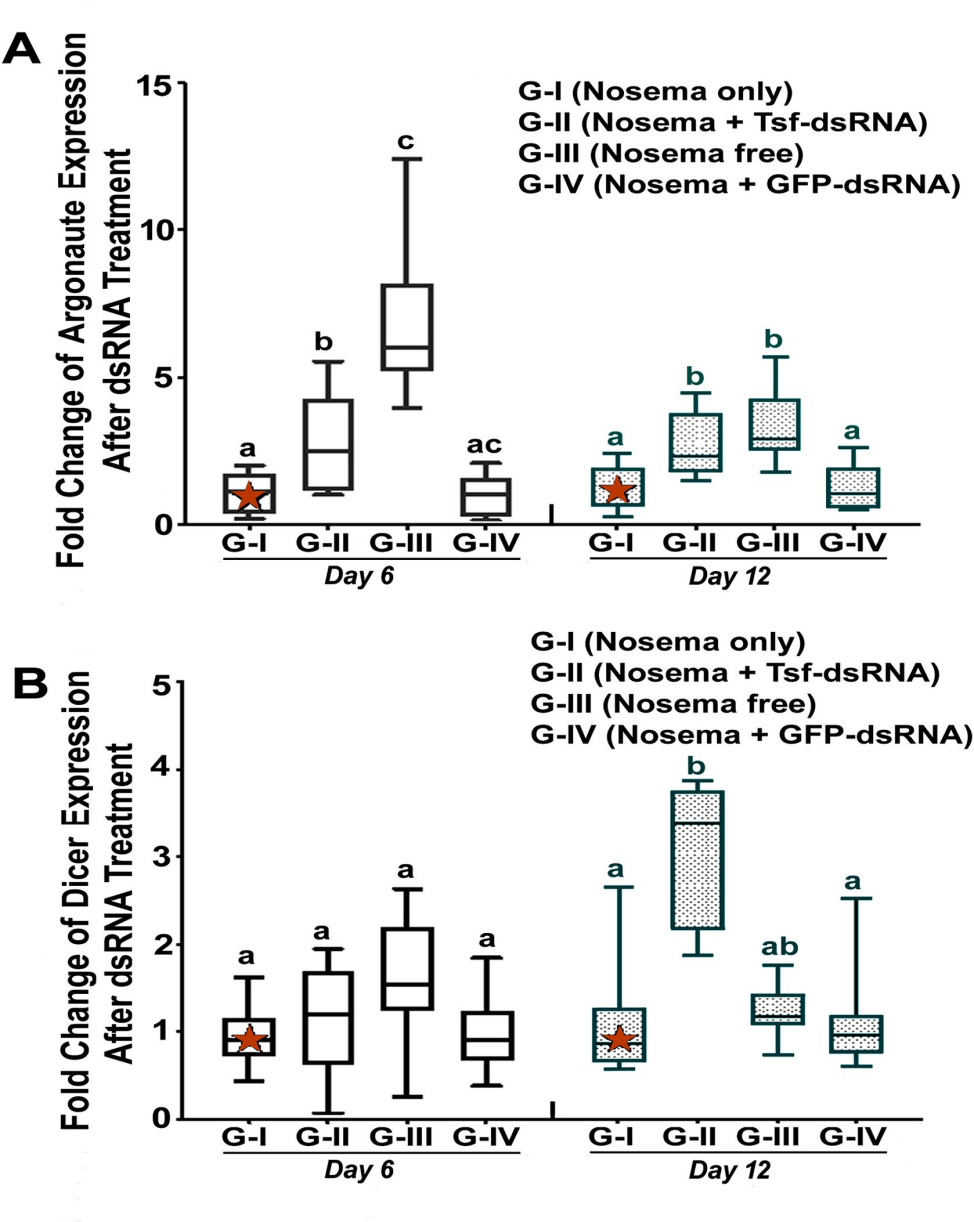
Transcript levels for RNA-induced silencing complex components. (A) *Argonaute*. (B) *Dicer*. The relative expression was expressed as an n-fold difference relative to the calibrator (marked by a star) by 2^–∆∆Ct^ method. A star denotes a calibrator and the different lower-case letters above bars indicate the statistically significant difference among different groups (*P* ≤ 0.05, ANOVA and Tukey´s tests).

The ingestion of AmTsf -dsRNA treatment was found to lead to not only target-specific silencing but also to a non-specific boost to transcripts which are core components of the RNAi signaling pathway, Dicer and Argonaute. The expression levels of Dicer and Argonaute in Group II were found to be significantly upregulated on day 12 post-treatment, compared to Group I and Group III (**Fig 10**) [Argonaute, 6 day: P = 0,063, F(3,30) = 3,065, One-way ANOVA and Tukey's multiple comparisons test G-I vs. G-II P = 0,8573, G-II vs. G-IV P = 0,7801. 12 day: P<0.0001, F(3,26) = 67,99, One-way ANOVA and Tukey's multiple comparisons test, G-I vs. G-II, p<0.0001, G-II vs. G-III, p<0.0001, G-II vs. G-IV, P <0,0001; Dicer 6 day: P = 0,0525, F(4, 37) = 2,589, One-way ANOVA and Tukey's multiple comparisons test G-I vs. G-II P>0,9999, G-II vs. G-IV P = 0,0961. 12 day: p<0.0001, F(3,32) = 20.68, One-way ANOVA and Tukey's multiple comparisons test G-I vs. G-II P<0.0001, G-II vs. G-III P<0.0001, G-II vs. G-IV P<0,0001].

### Increased total iron, as a result of AmTsf -dsRNA treatment, contributed to improved longevity

**Fig 11:**
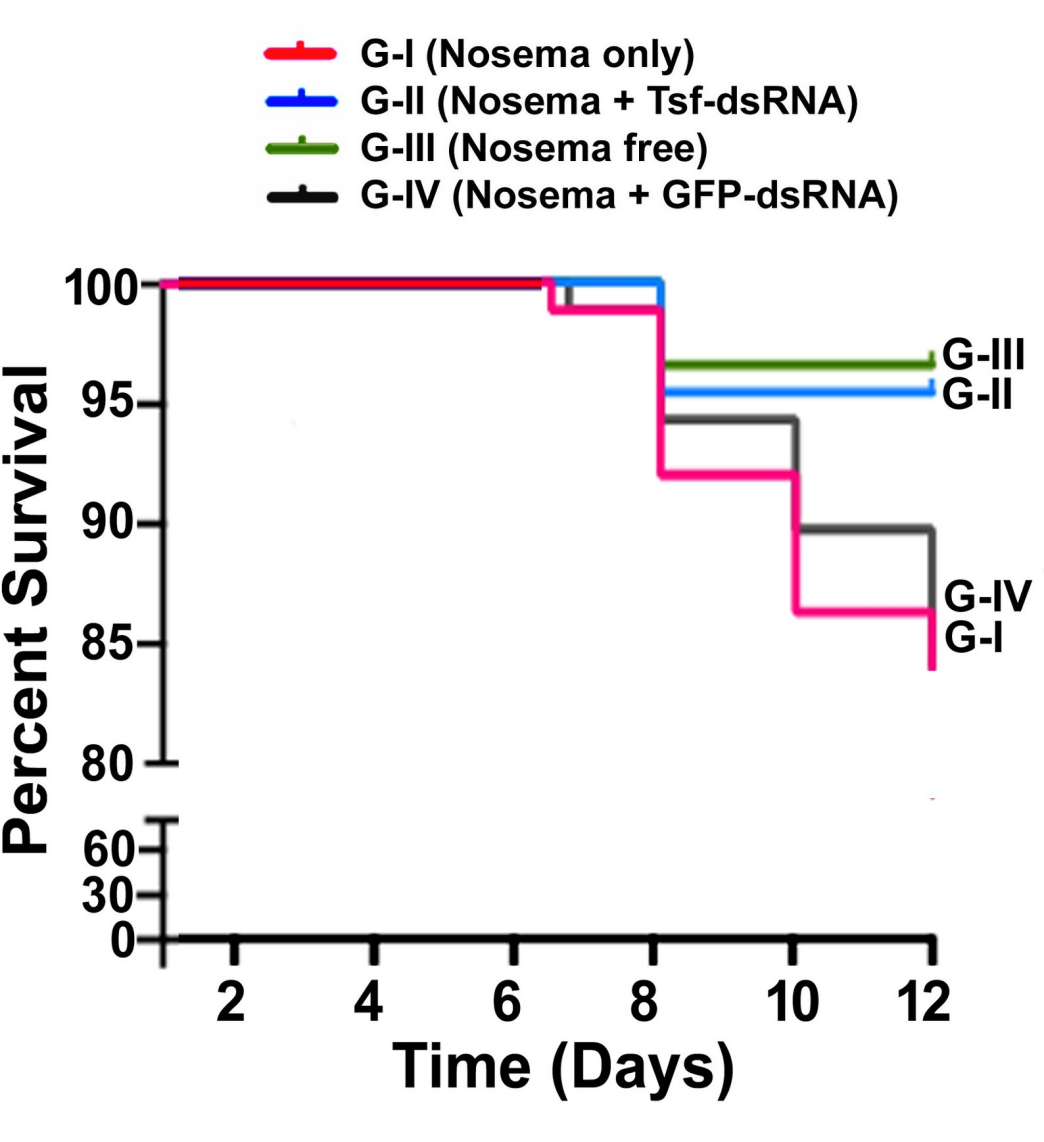
Kaplan–Meier survival curves for the three experimental groups (N = 90 bees/group). Data represent percentage of survival for the four groups over 12 days of experimental observation. Group I: *Nosema*-infected bees (infected/untreated); Group II: *Nosema*-infected bees + *AmTsf* -dsRNA treatment (infected/treated); Group III: Healthy bees (uninfected/untreated) and Group IV: Nosema-infected bees + GFP-dsRNA. Knockdown of the honey bee *transferrin* gene reduced the incidence of death (*p*< 0.05 by *Wilcoxon* test <*p = 0*.*0041>* and Log-rank test <*P* = 0.0033>).

Cumulative mortality was measured to evaluate the phenotypic effects of increased iron availability as a result of AmTsf-dsRNA treatment on *N*. *ceranae* infected honey bees. As shown in **Fig 11**, the percentage of survival among the three groups was significantly different at day 12 post treatment (Kaplan-Meier method (*P<0*.*05*), and log-rank (*P* = 0.0033) and Wilcoxon (*P = 0*.*0041*) tests were performed). *Nosema*-infected bees without RNAi treatment had the highest mortality among the three groups, while Group II, which received AmTsf-dsRNA treatment, had significantly improved survivorship and displayed a cumulative mortality curve similar to the healthy bees of Group III.

## Discussion

The iron tug-of-war between host and pathogen is a central battlefield at the host-pathogen interface which determines the outcome of an infection, however, has not been explored in honey bees. We conducted a study to investigate the impact of *N*. *ceranae* infection on iron homeostasis in honey bees and the results of the present study indicate the following two key findings. First, *N*. *ceranae* infection was found to cause honey bee iron deficiency and elevated expression of transferrin, an iron binding and transporting protein, implying that *N*. *ceranae* has evolved a mechanism to scavenge iron from the host. Second, the suppressed expression level of gene encoding transferrin (AmTsf) via RNAi could alleviated iron loss, enhanced immunity, and improved survival of the infected bees. The intriguing multifunctionality of transferrin illustrated in this study is a significant contribution to the existing body of literature concerning iron homeostasis in insects. The uncovered functional role of transferrin on iron homeostasis, pathogen growth and honey bee’s ability to mount immune responses may hold the key for the development of novel strategies to treat or prevent diseases in honey bees.

Previous studies have shown that the intensity of *N*. *ceranae* infection varies significantly among different age cohorts of honey bees in bee colonies and that foraging bees had a higher level of *N*. *ceranae* infection when compared to nurse bees [43, 83, 84]. Our study displayed these infection dynamics at the colony level and showed that the relative transcript abundance of *N*. *ceranae*16S rRNA was significantly higher in foraging bees than in nurse bees from the same colonies. This phenomenon that *N*. *ceranae* infection in foraging bees was considerably higher than in nurse bees may be a result of a build-up of *N*. *ceranae* load with age increasing over the time course of transition from nurse bees to foragers following initial infection at the earlier nursing stage. The high level of *N*. *ceranae* infection in honey bees has been reported to be associated with serious pathophysiological consequences including disruption of orientation performance, alteration of metabolism and nutrition, suppression of immune response, reduction of productivity and longevity of worker bees, and eventually possible collapse of the entire colony [23, 31, 85–88].

Microsporidia as “the master parasites” [89] have evolved specific mechanisms for interacting with their hosts, acquiring nutrients and metabolites from their hosts, and counteracting and evading their hosts’ immune system. Iron is an essential nutrient for host cells and pathogenic microbes alike. Our study showed that iron was deposited predominantly in the fat bodies of honey bees. This result supports the notion that insect fat body is the functional equivalent of the mammalian liver which is a major storage organ of iron. We found that *N*. *ceranae* parasitism could result in a significant reduction of the total body iron store, and that the decline of the total amount of iron was greater with an increase in *N*. *ceranae* load. While living organisms avoid an excess of free iron which can cause oxidative stress and result in a variety of ailments, there is growing scientific evidence that iron deficiency is a common pathology of iron homeostasis [90–93]. Iron-related pathogen-host interactions have been best characterized in bacteria and their mammalian hosts. For host defense against intracellular bacteria, a key strategy is to prevent the invading pathogens from acquiring the iron in a process termed nutritional immunity [94]. In this process, intestinal iron absorption is reduced and free iron is sequestered in the liver and other iron storage organs, thereby limiting iron availability to invading pathogens. However, prolonged iron sequestration as a defense mechanism not only restricts the iron supply to pathogens but also can limit the availability of iron to the host which can eventually result in alterations in the host’s immune function and increase the host’s susceptibility to infections [90, 93]. Our study provides the evidence that *N*. *ceranae* infection can lead to a significant iron loss in infected bees that signals an altered state of iron homeostasis. We conclude that a significant reduction of the total amount of iron store may also reflect the iron-withholding nutritional immunity exerted by the honey bee host.

To defy host nutritional immunity, intracellular pathogens have adopted methods to satisfy their own iron requirements by either synthesizing and secreting iron-chelating agents, siderophores, to scavenge iron from the intracellular environment or directly capturing iron from the iron-binding proteins ferritin or hemosiderin at the bacterial cell membrane [95, 96]. There is mounting evidence showing that most fungi produce siderophores, and that siderophores are critical virulence determinants for both animal- and plant-pathogenic fungi [97–99]. It is therefore entirely possible that the spore-forming fungus *N*. *ceranae* is able to produce siderophores to capture iron from infected hosts for its full virulence and survival. Further studies are needed to confirm the involvement of siderophores in *N*. *ceranae* pathogenesis and explore whether chelators siderophores can be used as novel targets for the pathogen-specific therapy in honey bees.

Compared to vertebrate animals, iron-related pathogen-host interactions in insects are poorly understood, and the molecular mechanisms involved in the regulation of iron homeostasis in insects remain mostly unknown. The principal regulator of systemic iron homeostasis in mammals is the liver peptide hepcidin, which is a negative regulator of iron absorption and recycling by binding to its receptor ferroportin which exports iron from cells into plasma [60]. Hepcidin deficiency was reported to cause iron overload while hepcidin excess leads to iron restriction [100]. While insects have a set of mammalian homologous proteins related to iron transport and storage, the sequencing and annotation of the *D*. *melanogaster* genome indicated that there are no recognized homologs to the mammalian hepcidin and ferroportin [60, 66]. Moreover, the exact function of certain mammalian homologous iron-regulatory proteins in insects remains unclear; in addition, some insect proteins may undergo functional divergence from their mammalian counterparts and employ different mechanisms for systemic iron regulation [70]. Further research is needed to determine whether the reduction of iron level due to *N*. *ceranae* infection in honey bees observed in our study is mediated by different cellular signaling pathways.

Transferrin is one of the key players of iron homeostasis, and is also involved in the innate immune functions. The ability of transferrin to bind iron with high affinity allows transferrin to maintain a low concentration of free iron in the body fluid, thereby limiting iron availability to invading pathogens. In the present study, the decrease in the total amount of iron in response to *N*. *ceranae* infection paralleled an increase in the expression level of AmTsf. The upregulation of AmTsf expression was tissue-specific and primarily restricted to the fat body of *A*. *mellifera*. The transcript level of AmTsf in the gut was much lower than that in the fat body. This result supports previous findings that the fat body is the primary site of insect transferrin-1 expression [67] and transferrin-1 is involved in trafficking iron from the gut to the fat body [68]. This result also ties well with the knowledge of mammals that transferrin is primarily expressed in the liver. The observed negative correlation between the level of AmTsf expression in the fat body and the total amount of iron in *N*. *ceranae* infected bees indicates a functional involvement of AmTsf in iron metabolism and suggests that higher expression of AmTsf may allow *N*. *ceranae* to plunder more iron from its host for growth and development. We, therefore, conclude that increased expression of transferrin is an indication of iron deficiency in disease-infected honey bees. The new insight about the functional role of transferrin in honey bee’s immune response to iron deficiency caused by microsporidia *Nosema* infection is a significant contribution to the existing body of literature concerning iron homeostasis in insects.

Most of the studies published so far on the role of transferrin as a mediator of antimicrobial immunity in the context of iron deficiency have focused on bacterial infections. The bacterial challenges have been reported to cause a significant increase in transferrin expression in vertebrates and invertebrates [90, 92, 93, 96, 101]. In addition to responding to the bacterial challenge, the iron-withholding strategy has also been reported as the first line of defense against fungi and viruses [90, 102]. Studies with insect species including *D*. *melanogaster*, *Manduca sexta*, and *A*. *aegypti* showed that the expression of transferrin, as an acute-phase protein, was upregulated in response to bacterial infection and suggested that the insect transferrin is involved in the immune function by participating in iron withholding, thus preventing bacterial growth [67, 101, 103]. While insect transferrin shares considerable sequence homology with mammalian serum transferrin, the exact mechanisms by which the expression of transferrin is activated upon pathogen infection are unclear. The transferrin receptor is a transmembrane protein for transferrin and participates in iron transport by internalizing the iron-bound transferrin through receptor-mediated endocytosis. The expression of transferrin and transferrin receptor is coordinately regulated by iron in mammalian systems [62]. Nevertheless, there is no recognized homolog to the mammalian transferrin receptor in insects [66], suggesting that transferrin may work in a different way in insects for transporting iron from hemolymph to host cells without the help of the transferrin receptor.

Despite the sequence and functionality of transferrin being preserved across different animals, the expression of transferrin in response to stress and infection varies in different species and its contribution to host defense is diverse and complex [101]. While the upregulation of transferrin expression in honey bees was activated by *N*. *ceranae* infection, it was unclear whether an elevated transferrin mRNA level would be beneficial or pose harm to the honey bee host. Our specific silencing of the expression of transferrin gene by RNA interference (RNAi) [104] showed that AmTsf*-*dsRNA could effectively suppress the expression of the AmTsf gene in *N*. *ceranae* infected bees. Interestingly, the adjustment of AmTsf expression by dsRNA treatment could lead to a reduction of *N*. *ceranae* disease as shown by a significantly decreased level of *N*. *ceranae* 16S rRNA transcription at the two time points post treatment. Consistent with the decrease in the level of *N*. *ceranae* 16S rRNA transcription, the RNA-induced silencing of AmTsf was found to ameliorate iron loss in *N*. *ceranae* infected bees, suggesting that iron deficiency could be monitored by adjusting Tsf activity. After 12 days of treatment, the overall improvement of health due to the knock down of AmTsf expression is evidenced by the fact that *N*. *ceranae* infected bees exhibited an extended lifespan and displayed a survivorship curve similar to the negative control group of healthy bees after dsRNA treatment. The same RNAi responses triggered by AmTsf-dsRNA were not noted in the bees of experimental group treated with GFP-derived dsRNA, confirming the target specificity of the employed RNAi method in this study. A previous study by Nunes et al 2013 [105] revealed that the expression of nearly 1,400 honey bee genes was altered in response to GFP-dsRNA. However, the effects of the interference mediated by GFP-dsRNA were not detected in our study. There was no significant difference between the Group I (Nosema only) and Group IV (Nosema+GFP-dsRNA) in the gene expression of N. ceranae 16S rRNA, transferrin, immune transcripts, cell apoptosis regulator, and RNAi pathway components. The discrepancy between our results and the findings from the previous report may be attributable to different amounts of GFP-dsRNA to induce RNAi effect (20 ng vs 500 ng). It was reported that use of relatively low concentrations of siRNA could lead to lower off-target effects [106, 107]. Given the positive effects of suppressing transferrin expression on the iron deficiency and the survival of *N*. *ceranae*-infected bees, it suggests that honey bees’ nutritional and immune responses to *Nosema* infection are interlinked and elevated transferrin mRNA level in honey bees could be detrimental. It also suggests that targeting microbial iron acquisition and suppressing aberrant expression of AmTsf using an RNA interference (RNAi) based method may hold the key for the development of novel therapeutic strategies for treating and preventing *Nosema* disease in honey bees.

RNAi is a remarkable process used to knock down the expression of a targeted gene and has provided unique opportunities in combating diseases caused by pathogens in a wide range of organisms including honey bees [108–111]. The potential of RNAi as a therapy for controlling *Nosema* disease in honey bees has been explored through two different angles under laboratory conditions. First, the silencing of *N*. *ceranae* virulence genes including polar tube protein 3 (PTP3), ATP/ADP transporter, and Dicer demonstrated that the transcript levels of the target genes and *Nosema* spore loads decrease significantly when honey bees ingest dsRNA specific to the target genes [112–114]. Secondly, RNAi-mediated knockdown of a host gene, namely naked cuticle (nkd), a negative regulator of host immunity which was up-regulated by *N*. *ceranae* infection, showed a significant reduction of *Nosema* spore loads, enhancement of immune expression, and extension of lifespan in *N*. *ceranae* infected bees [115]. While the results of these studies clearly demonstrate that RNAi-based therapeutics hold great promise for the effective treatment of honey bee diseases, there are still obstacles to overcome for large-scale therapeutic applications. Potential undesirable effects of RNAi include off-target effects, innate immune stimulation, and effective delivery into target cells and tissues which must be carefully tested and validated before using this approach [116]. Various strategies have been explored and developed to address the hurdles facing RNAi-based therapeutics [106]. For example, chemical modifications of siRNA and miRNA analogs, sequence optimization and identification of the lowest siRNA concentration that gives a specific knockdown of the target mRNA have been shown to be effective approaches for improving the specificity and minimizing the risk of adverse effects of RNAi-based therapeutics. Nanotechnology as a delivery strategy has been developed to increase stability and facilitate delivery of RNAi-based drugs. With the availability of Alnylam’s patisiran, the first ever FDA- and European Commission-approved RNAi therapy after 20 years in research and development, for the human disease treatment, the future of RNAi-based therapy as a safe and cost-effective treatment in the beekeeping industry looks promising.

The Toll pathway is one of the major regulators of the immune response in *A*. *mellifera* and controls the expression of genes that encode the antimicrobial peptides (AMPs) that are synthesized in fat bodies and released into the hemolymph. [117]. The expression of two AMPs *apidaecin* and *abaecin* was found to be significantly suppressed in *N*. *ceranae* infected bees in the present study and previous reports [32, 37, 113]. The expression level of *apidaecin* and *abaecin* was enhanced after *AmTsf* -dsRNA treatment. Meanwhile, BIRC5, a gene encoding an apoptosis inhibitor protein (IAP) was found to be overexpressed along with the upregulation of transferrin expression. Apoptotic cell death regulates cell proliferation and is an integral part of innate immune function. IAP proteins are a group of negative regulators of apoptotic pathways and downstream innate immune signaling of pattern recognition receptors (PRRs), such as the Toll-like receptors (TLRs) [118]. The overexpression of an IAP was reversed by the silencing of transferrin by dsRNA molecules. Similarly, the AmTsf -dsRNA treatment was also found to lead to a non-specific boost to the abundance of two transcripts, Dicer and Argonaute, which are core components of the RNAi signaling pathway. Our study provided novel evidence that overexpression of transferrin due to *N*. *ceranae* infection could suppress the honey bee’s immune response, thereby making immunodeficient honey bees more susceptible to the disease infection. The dual-functional outcomes of RNAi in our study that effectively knocked down the expression of target gene AmTsf and also elicited innate immune responses in honey bees indicate the connection of immunity and iron hemostasis and points to a potent therapeutic strategy for limiting *Nosema* disease in honey bees.
